# Hydrophilicity Improvement of Polymer Surfaces Induced by Simultaneous Nuclear Transmutation and Oxidation Effects Using High-Energy and Low-Fluence Helium Ion Beam Irradiation

**DOI:** 10.3390/polym12122770

**Published:** 2020-11-24

**Authors:** Jung Woo Kim, Seung Hwa Yoo, Young Bae Kong, Sung Oh Cho, Eun Je Lee

**Affiliations:** 1 Advanced Radiation Technology Institute, Korea Atomic Energy Research Institute, 29 Geumgu-gil, Jeongeup-si, Jeollabuk-do 56212, Korea; kjw924@kaeri.re.kr (J.W.K.); ybkong@kaeri.re.kr (Y.B.K.); 2 Department of Nuclear and Quantum Engineering, Korea Advanced Institute of Science and Technology, 291 Daehak-ro, Yuseong-gu, Daejeon 34141, Korea; socho@kaist.ac.kr; 3 Department of Quantum System Engineering, College of Engineering, Jeonbuk National University, Jeonju 54896, Korea

**Keywords:** high energy low fluence, helium ion, irradiation, polymer, surface wettability, water contact angle, nuclear transmutation, oxidation

## Abstract

Two commodity polymers, polystyrene (PS) and high-density polyethylene (HDPE), were irradiated by high-energy He ion beams at low fluence to examine the wettability changes at different fluences. The water contact angles of the PS and HDPE surfaces were reduced from 78.3° to 46.7° and 81.5° to 58.5°, respectively, upon increasing the fluence from 0 to 1 × 10^13^ He^2+^/cm^2^ for irradiation durations ≤4 min. Surface analyses were performed to investigate these wettability changes. Surface texture evaluations via scanning electron and atomic force microscopies indicated non-remarkable changes by irradiation. However, the chemical structures of the irradiated polymer surfaces were notable. The high-energy He ions induced nuclear transmutation of C to N, leading to C–N bond formation in the polymer chains. Further, C–O and C=O bonds were formed during irradiation in air because of polymer oxidation. Finally, amide and ester groups were generated by irradiation. These polar groups improved hydrophilicity by increasing surface energies. Experiments with other polymers can further elucidate the correlation between polymer structure and surface wettability changes due to high-energy low-fluence He ion irradiation. This method can realize simple and effective utilization of commercial cyclotrons to tailor polymer surfaces without compromising surface texture and mechanical integrity.

## 1. Introduction

Engineering the surface wettability of solids has been intensively studied to improve the adhesion of coating/printing/painting, lower friction between different surfaces, and achieve functional surfaces that exhibit self-cleaning/anti-icing/anti-fingerprint properties. It is well known that the wettability of a solid surface is mainly governed by its physical texture (e.g., roughness, hierarchy, and pore structure; Wenzel and Cassie–Baxter models) and chemical composition (e.g., functional groups, polarity, and charge state). The liquid (e.g., water) contact angle on these solid surfaces is determined by the force balance between adhesive and cohesive forces. The cohesive force (surface tension) is liquid-dependent; however, the adhesive force can be controlled by both the solid and liquid phases. Therefore, many efforts have been made to manipulate the surface properties of solids to increase their adhesive force and subsequently improve their surface wettability.

Several methods have been successfully exploited to increase surface wettability by treating solid surfaces both chemically and physically. These routes alter the surface chemistry, physical texture, or both of the solid surface. Self-assembled monolayers (e.g., sol-gel method and phase separation) are typical chemical routes used to functionalize or oxidize solid surfaces by introducing hydrophilic groups (surface chemistry) [1,2,3,4]. These routes can also affect the surface texture by etching the surface. These chemical routes are effective for engineering surface wettability; however, they usually require precise and controlled conditions for time-consuming reactions. Furthermore, they are less eco-friendly and use toxic chemical agents/solvents, generate waste chemicals (depleted solution, washing solvent), and should consider the economic feasibility of commercialization because of their cost and time consumption. However, physical routes have advantages in this regard. Treatments using plasma, ionizing radiation, thermal annealing, corona discharge, etc., are effective in increasing the surface roughness and introducing hydrophilic groups onto solid surfaces to increase the surface wettability [5,6,7,8,9,10,11]. Low-energy particle (mostly ions) bombardment is usually accompanied by these methods, which effectively alters the surface properties; however, there is a compromise in the mechanical strength because of the etching of the solid.

Various materials, such as metal (e.g., tungsten, iron, nickel, and alloys), carbon (e.g., diamond [12] and graphene [13]), semiconductors (e.g., silicon [14,15], lead halide [16]), and composites, have been irradiated by low-energy high-fluence He ions to alter their surface properties. Among these, a number of studies have conducted He ion irradiation with polymers as the target material. Li et al. recently studied low-energy high-fluence He ion irradiation (ion implantation) of Kapton, polyethylene terephthalate (PET), polytetrafluoroethylene (PTFE), and fluorinated ethylene propylene (FEP) for triboelectric nanogenerators. He ions with 50 keV energy and 1 × 10^16^ He^2+^/cm^2^ fluence were used to modify the surface properties of these polymers by ion implantation. They reported that the electron-donating capability of the Kapton was enhanced through the tuning of chemical structures and functional groups [17]. Gelamo et al. conducted He ion irradiation (energy: 170 keV, fluence: 1 × 10^14^–1 × 10^16^ He^2+^/cm^2^) of polymer films deposited from TMS-Ar plasmas and studied the changes in polymer structure and hardness. They observed the depletion of C–H and Si –H and formation of O–H and Si–O with increasing hardness. They concluded that the polymer structure transformed into a silicon oxycarbide network by irradiation [18]. Park et al. conducted a dual-ion beam irradiation (including He ions, energy: 70 keV, fluence: 3 × 10^16^ to 1 × 10^17^ ions/cm^2^) of a polymer blend to modify the optical properties of the coatings and improve the adhesion between the polymer surface and coating. By employing dual-ion beams, they effectively mitigated the highly crosslinked brittle layer that emerged from heavy single ion irradiation [19].

In this study, polystyrene (PS) and high-density polyethylene (HDPE) sheets were irradiated by high-energy low-fluence He ion beams to study their changes in surface wettability. These polymers are the commodity plastics most widely used in daily lives. In particular, PS is known as a polymer highly resistant to ionizing radiation; hence, its property changes upon irradiation are slightly less than those of other polymers. Surface wettability measurements of irradiated PS and HDPE were evaluated at different fluences. Various surface analyses of the irradiated polymers were conducted to elucidate the wettability change caused by irradiation.

## 2. Materials and Methods

### 2.1. Materials and Sample Preparation

PS and HDPE sheets with thicknesses of 1.2 and 1.0 mm, respectively, were purchased from Goodfellow Cambridge Ltd., Cambridge, UK. These sheets were cut into 25 mm × 25 mm pieces for the irradiation experiments.

### 2.2. He Ion Beam Irradiation

The as-prepared polymer samples were irradiated by a He ion (also called alpha (α) particle) beam with energy 20 MeV and current 50 nA using the MC-50 Cyclotron installed at the Korea Institute of Radiological and Medical Sciences (KIRAMS). The total fluence was controlled to be 3 × 10^12^, 5 × 10^12^ and 1 × 10^13^ He^2+^/cm^2^ by adjusting the irradiation duration to 1.2, 2 and 4 min, respectively. The irradiation durations used in this study are far shorter than those discussed in the literature despite the low current. The polymer samples were exposed to the ion beam within a diameter of 20 mm. The gamma spectra of the irradiated polymer samples were measured using a high-purity Germanium (HPGE) detector (ORTEC, GEM10P4, Oak Ridge, TN, USA) connected to a multichannel analyzer (MCA) system. The ion trajectories within the polymers were simulated using the SRIM code [20]. The displacement energies were set to 28 and 10 eV for C and H, respectively, and the densities of the polymers were 1.16 g/cm^3^ for PS and 0.94 g/cm^3^ for HDPE.

### 2.3. Characterization

The surface morphologies of the pristine and irradiated polymer samples were observed using field-emission scanning electron microscopy (FE-SEM; SU5000, Hitachi, Tokyo, Japan) and atomic force microscopy (AFM; XE-70, Park System, Suwon, Republic of Korea). Non-contact mode AFM was operated using high-resonant-frequency cantilevers (~330 kHz) with PPP-NCHR tips. Roughness analysis was performed on pristine and irradiated polymer surfaces using the commercial surface analytical software XEI. Mean values of root mean square (RMS) roughness were determined by at least five scanning areas (4 μm × 4 μm squares). The chemical structures of each polymer sample surface were characterized by X-ray photoelectron spectroscopy (XPS, K-alpha, Thermo VG Scientific, Waltham, MA, USA) over a 400 μm diameter analysis area. The XPS data acquisition was performed using an automated monochromatic X-ray source (Al K-alpha) operated at 12 kV and 3 mA. Survey spectra and high-resolution spectra were obtained using pass energies of 200 eV and 50 eV, respectively. A Fourier-transform infrared spectrometer (FT-IR; Nicolet IS50, Thermo Fisher Scientific Instrument, Waltham, MA, USA) was used in attenuated total reflection (ATR) mode. The pristine and irradiated polymer samples were scanned in the IR wavenumber region of 4000–400 cm^−1^. The wetting properties of the pristine and irradiated polymer sample surfaces were characterized using a contact angle (CA) measurement system (SEO Co., Ltd., Suwon, Republic of Korea, Phoenix 300 Plus). A water droplet of 4 μL was used for each surface, and measurements were performed at five different positions for each sample to estimate the water CA.

## 3. Results and Discussion

**Figure 1:**
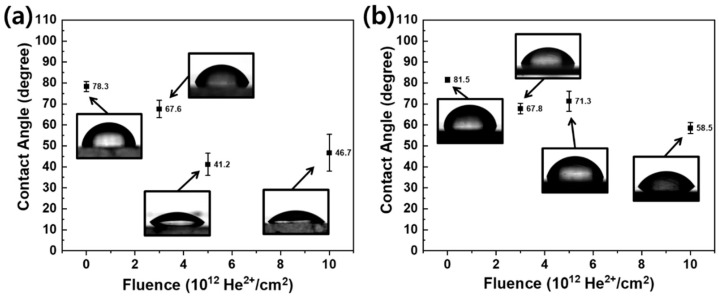
Water CA plots of (**a**) PS and (**b**) HDPE irradiated at various fluences.

Figure 1 displays the changes in the water CA of PS and HDPE with increasing fluence of He ion irradiation. In general, the water CA of both polymers gradually and significantly decreased with increasing fluence. The average CA of pristine PS and HDPE was 78.3° and 81.5°, respectively, which significantly decreased to 46.7° and 58.5° at 1 × 10^13^ He^2+^/cm^2^. The fluence used in this study was extremely low compared to that in other studies (usually 10^15^–10^16^ He^2+^/cm^2^) [17,21,22,23,24,25], which indicates that He ion irradiation effectively modifies the surface wettability of polymers. The enhancement of hydrophilicity was relatively prominent for PS compared to that for HDPE. To elucidate the origin of these enhancements, various analyses were conducted to characterize the surface properties of the irradiated polymers.

**Figure 2:**
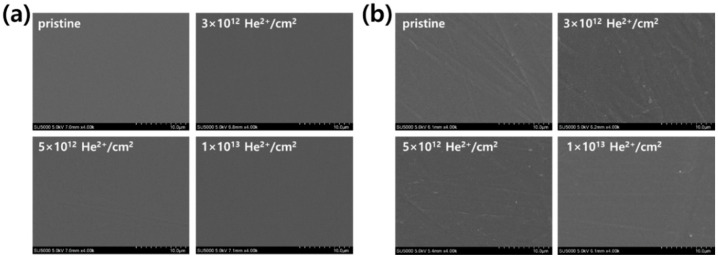
Top surface FE-SEM images of (**a**) PS and (**b**) HDPE irradiated at various fluences.

**Figure 3:**
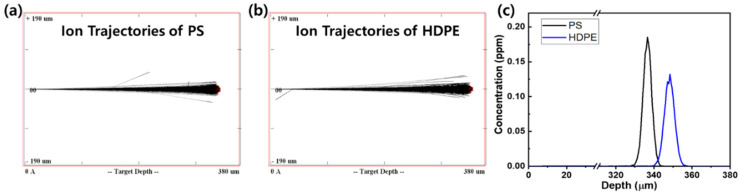
Ion trajectories of a 20 MeV He ion beam irradiated onto (**a**) PS, (**b**) HDPE and (**c**) He ion depth-dependent concentration profile simulated using SRIM code.

**Figure 4:**
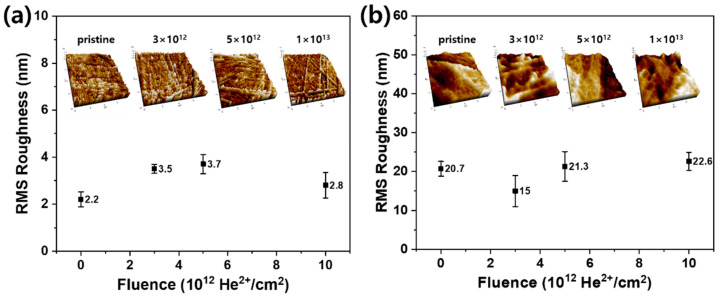
RMS roughness and corresponding AFM images of (**a**) PS and (**b**) HDPE surfaces irradiated at various fluences.

First, the surface morphologies of the pristine and He ion-irradiated polymers were observed by FE-SEM, as shown in Figure 2. Despite irradiation, the polymer surfaces showed unnoticeable changes for all fluences. This observation was attributed to the high energy and low fluence used in this study, which did not cause severe damage or defects on the polymer surfaces. The simulated trajectories of 20 MeV He ions in PS and HDPE are shown in Figure 3. The high-energy He ions easily penetrated the polymer sheets, so the ions were not implanted at the polymer surface. Most He ions were stopped below 300–400 µm surface depth. As a result, the polymer surface was not physically damaged in this study. The surface topography at higher magnification was measured by AFM, and its surface roughness was estimated for different fluences (Figure 4). In the case of PS, the average RMS roughness ranged from 2.2 to 3.7 nm at different fluences, indicating that a small variation in roughness was caused by irradiation (Figure 4a). In a similar manner, small variations in roughness were also observed for HDPE at different fluences (Figure 4b). Therefore, it was concluded that the surface roughness was not considerably changed by irradiation of PS and HDPE at the low fluences used in this study.

**Figure 5:**
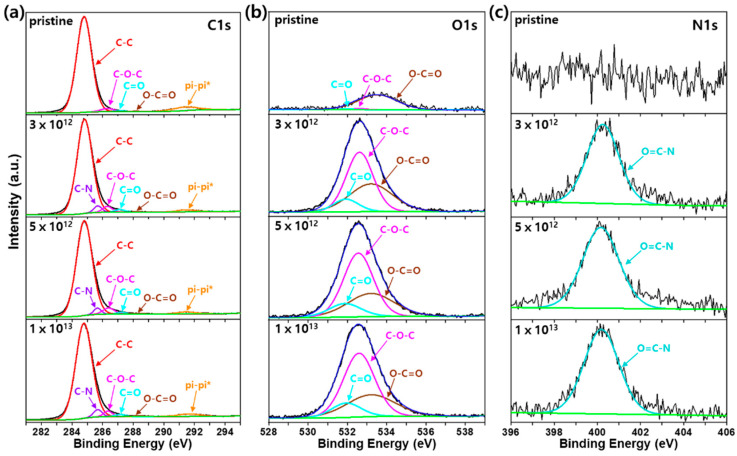
XPS deconvolution spectra of (**a**) C1s, (**b**) O1s, and (**c**) N1s for irradiated PS at various fluences.

**Figure 6:**
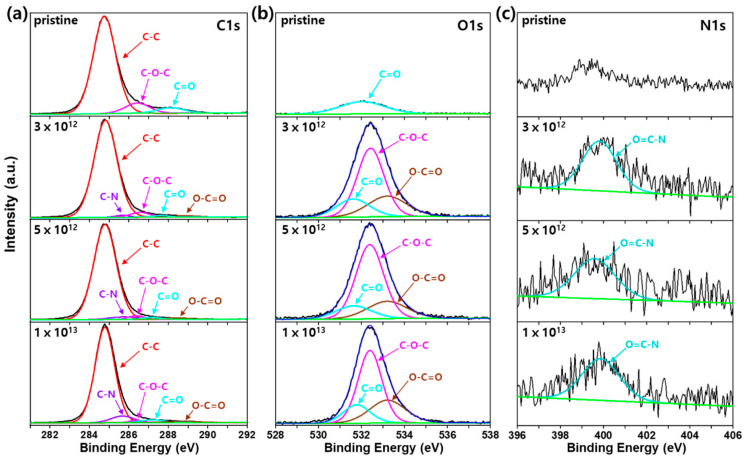
XPS deconvolution spectra of (**a**) C1s, (**b**) O1s, and (**c**) N1s for irradiated HDPE at various fluences.

Figure 5 and Figure 6 show the XPS deconvolution spectra of pristine and irradiated PS and HDPE, respectively, at different fluences. Significant changes in the atom-atom bond structure of the irradiated polymers were evaluated by XPS analysis, which produced contrary results to those of morphological changes, as discussed above. First, the C–C (284.8 eV) bond was the main component of both pristine polymers (Figure 5a and Figure 6a) with a π–π* shake up satellite peak for PS (Figure 5a).

Furthermore, oxygen (O) containing bonds, such as C–O–C, C=O and O–C=O for PS (Figure 5a) and C–O–C, C=O for HDPE (Figure 6a), were observed in the pristine polymer samples. We believe that these oxygens were introduced during the polymer processing of the sheets used in this study. These O-containing bonds were further verified by analyzing the O1s spectra of pristine PS and HDPE (Figure 5b and Figure 6b). After He ion irradiation, the atomic percentage of O gradually increased from 1.38 to 14.48% and 6.28 to 9.56% for PS and HDPE, respectively, as the fluence increased from 0 to 1 × 10^13^ He^2+^/cm^2^. Surprisingly, a considerable amount of nitrogen (N) was detected after irradiation of PS and HDPE. The atomic percentage of N was gradually increased from 0 to 1.95% and 0 to 0.31% for PS and HDPE, respectively, with increasing fluence from 0 to 1 × 10^13^ He^2+^/cm^2^. Further N1s XPS analysis revealed that the O=C–N (400.2 eV) bond structure was formed (Figure 5c and Figure 6c). Therefore, it is clear that nitrogen was generated by irradiation of both polymers.

Despite the low fluence used in this study, bombardment of high-energy He ions on the polymers could physically and chemically affect their constituent atoms and atom–atom bonds. In general, nuclear reactions can be induced if the energy of the incident ion is higher than the threshold energy of several nuclear reactions, as listed in Table 1 for 20 MeV He ions. Carbon is the most abundant atom in polymers, and C-12 (98.9%) and C-13 (1.1%) are stable isotopes that exist in nature. Based on Table 1, C-12 and C-13 can eventually transform to N-15 by nuclear transmutation reaction induced by 20 MeV He ion irradiation, which is above the threshold energy of each reaction. Furthermore, O-16 could be produced from the transmutation of C-13. Explanation of the evidence that reactions (1) and (5) certainly occurred in irradiated polymers is given by the gamma spectrum in Figure S1 in the supporting information.

Along with the nuclear reaction induced by high-energy He ion irradiation, oxidation of the polymer could also occur when exposed to air during irradiation. It is well known that when polymers are irradiated by ionizing radiation, various polymer radical species can be generated and subsequently react with oxygen in air to form various oxidized species within the polymer [26,27,28,29,30,31]. In addition, high-energy He ion beams can ionize oxygen and nitrogen molecules found in air, and these ions can subsequently react with the irradiated polymer surfaces [32,33,34].

**Figure 7:**
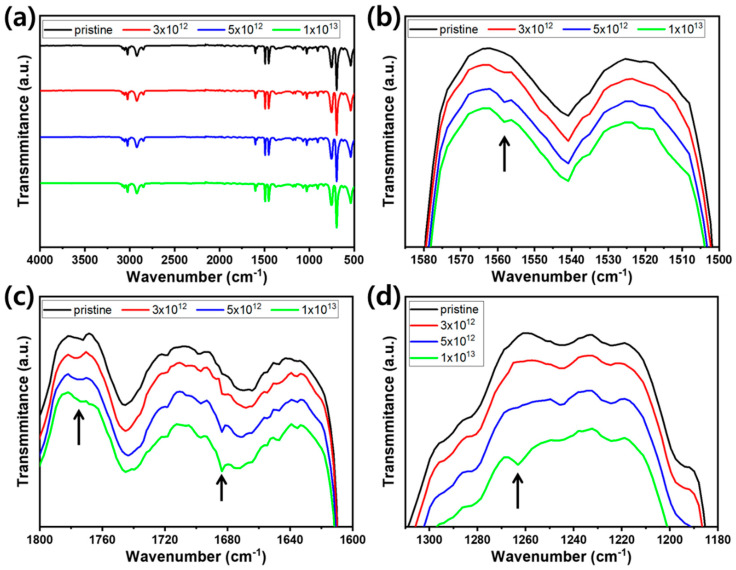
FT-IR spectra of pristine and irradiated PS in the (**a**) 4000–500 cm^−1^, (**b**) 1590–1500 cm^−1^, (**c**) 1800–1600 cm^−1^, and (**d**) 1320–1180 cm^−1^ wavenumber ranges.

**Figure 8:**
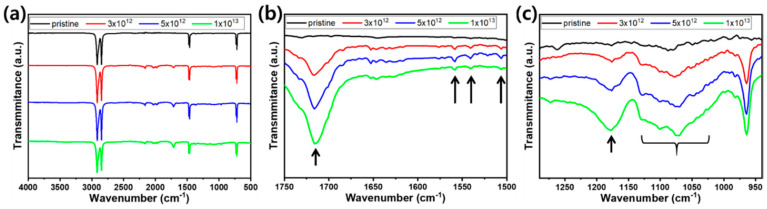
FT-IR spectra of pristine and irradiated HDPE in (**a**) 4000–500 cm^−1^, (**b**) 1750–1500 cm^−1^, and (**c**) 1290–940 cm^−1^ wavenumber ranges.

The irradiated PS and HDPE were further analyzed using FT-IR spectroscopy. The FT-IR spectra of irradiated PS and HDPE at various fluences are displayed in Figure 7 and Figure 8, respectively. In the case of PS, typical vibration modes of aromatic C—H stretching (3060.8 and 3026.0 cm^−1^), aromatic C=C stretching (1600.4, 1492.2 and 1452.0 cm^−1^), C—H out-of-plane bending (756.0 and 698.2 cm^−1^), methylene associated vibrations (2921.9 and 2848.6 cm^−1^), and various overtones (2000–1650 cm^−1^) were observed. Along with these vibration modes, additional modes were observable, which might have originated from O-containing groups within the pristine PS samples, which would be consistent with the XPS analysis. Based on their wavenumbers, it is suspected that various types of oxidized species, such as ether (C-O stretching: 1150 cm^−1^) and ester (C–O stretching: 1182 and 1310 cm^−1^), were present in the pristine PS samples used in this study. Contrary to the XPS analysis, it was difficult to observe the N-containing vibration modes in the irradiated PS samples in the FT-IR analysis. Vibration modes, such as amide C=O stretching (~1680–1630 cm^−1^), amide C—N stretching (~1550–1500 cm^−1^), NH & NH_2_ stretching (~3350, 3180 and 3300 cm^−1^) and bending (~1640–1500 cm^−1^), amine N—H bending (1650–1580 cm^−1^), aromatic amine C—N stretching (1342–1266 cm^−1^), and amine N—H stretching (3350–3310 cm^−1^), could be expected to be detected in FT-IR from the formation of O=C–N by PS irradiation. We suppose that the difficulty in observing the abovementioned modes was because of the low content of C–N bonds generated by irradiation (N less than 2 at% at 10^13^ He^2+^/cm^2^) and overlapping with the existing vibration modes, such as aromatic C=C stretching (1600.4 cm^−1^) and ester C—O stretching (1310 cm^−1^). Only minute signals detected at 1683, 1558 and 1264 cm^−1^, which were assumed to be amide C=O stretching, amide C—N stretching, and aromatic amine C—N stretching, respectively, developed by irradiation. The NH and NH_2_ vibration modes were undetectable, possibly because of the very low amount existing in the irradiated PS. Meanwhile, a shoulder peak at ~1770 cm^−1^ developed as a result of the formation of C=O stretching mode after irradiation of PS, as shown in Figure 7c.

In the case of HDPE, typical vibration modes of CH_2_ stretching (2914 and 2846 cm^−1^), CH_2_ scissoring and wagging (1462 and 1367 cm^−1^), CCH bending (1367 and 1262 cm^−1^), C–C stretching (1188 cm^−1^), and CH_2_ rocking (719 cm^-1^) were observed. Similar to pristine PS, various O-containing groups, such as ether (C–O stretching: 1263 and 1087 cm^−1^) and ester (C–O stretching: 1304 cm^−1^ and C=O stretching: 1730 cm^−1^), were thought to be present in the pristine HDPE samples used in this study. After irradiation, the most prominent change was the development of a vibration mode at 1735–1715 cm^−1^, which was assigned to ester C=O (Figure 8b). Furthermore, vibration modes at ~1176 cm^−1^ and 1150–1055 cm^−1^, assigned to ester C–O and ether C–O stretching, respectively, were developed by irradiation. These were attributed to the oxidation of HDPE during irradiation in air. In addition to oxidation, minute signals, which are assumed to originate from amide C–N stretching (1541 cm^−1^), amide N–H bending (1588 cm^−1^) and N–H stretching (1506 cm^−1^), were detected after irradiation as a result of the nuclear transmutation of C–C to C–N.

Based on our analysis, we suggest that the hydrophilicity of polymers was improved by the combined effects of nuclear transmutation and oxidation induced by high-energy He ion irradiation. Despite the high-energy ion bombardment of polymers, there was no macroscopic morphological change in their surface to affect the wettability. Slight microscopic changes in surface roughness were observed at different fluences; however, this could not solely explain the steady improvement of hydrophilicity as fluence was increased. Meanwhile, the chemical structures of the polymer surfaces were noticeably modified by irradiation despite the low fluence. The high energy of the He ion (as 20 MeV) could induce several nuclear reactions to transform C into N and therefore generate C–N bonds from abundant C–C bonds in polymers. Furthermore, 20 MeV He ions are ionizing radiation, which can easily produce polymer radicals. These polymer radicals could react with oxygen and finally form oxidized species during irradiation and storage in air. As a result, various O-containing groups were developed by prolonged irradiation. In conclusion, N– and O-containing (amide and ester) groups were formed in PS and HDPE by irradiation. These polar groups could effectively improve the wettability towards hydrophilicity by increasing the surface energy.

## 4. Conclusions

In this study, PS and HPDE sheets were irradiated by a high-energy but low-fluence He ion beam, and the surface wettability was evaluated through water CA measurements. The water CA could be gradually and effectively lowered by simply increasing the fluence. It was found that the water CA could be lowered to 46.7° and 58.5° at 1 × 10^13^ He^2+^/cm^2^ for PS and HDPE, respectively. The morphology and roughness of the polymer surfaces were evaluated by SEM and AFM, which indicated non-remarkable changes by irradiation. However, the chemical structure of the irradiated polymer surfaces showed several noteworthy features. Owing to the high energy of He ions, a certain amount of C was transformed to N by nuclear reaction. This led to the formation of C–N bonds in the polymer chains, which are abundant in C–C bonds. In addition, C–O and C=O bonds were formed during irradiation in air because of the oxidation of polymers. Finally, amide and ester groups were generated in PS and HDPE through irradiation. Therefore, the polar groups formed in non-polar PS and HDPE improved the hydrophilicity by increasing the surface energy. The advantage of the high-energy low-fluence irradiation method is that it can be conducted using commercial cyclotrons with very short irradiation durations (4 min for 1 × 10^13^ He^2+^/cm^2^ fluence). Further experiments with other polymers would be promising to study the correlation between polymer structure and surface wettability change by high-energy low-fluence He ion irradiation. We believe that this method could be a simple, effective, and practical route to tailor polymer surfaces without compromising the surface texture and mechanical integrity.

## Figures and Tables

**Table 1:** Most possible nuclear reactions induced by 20 MeV He ion beam irradiation for polymers.

#	Nuclear Reaction	Threshold Energy (MeV)	Decay Mode, Half-life
(1)	^12^C (α, n)^15^O → ^15^N	11.338	β^+^, 122.24 s
(2)	^12^C (α, p)^15^N	0	
(3)	^13^C (α, n)^16^O	0	
(4)	^13^C (α, p)^16^N → ^16^O	9.708	β^−^, 7.13 s
(5)	^13^C (α, 2n)^15^O → ^15^N	17.588	β^+^, 122.24 s
(6)	^13^C (α, np)^15^N	12.962	

## Supplementary Materials

The following are available online at https://www.mdpi.com/2073-4360/12/12/2770/s1, Figure S1: Measured gamma spectrum of a polymer irradiated with a 20 MeV helium ion beam.
