# Supplementary material for: Hydrophilicity Improvement of Polymer Surfaces Induced by Simultaneous Nuclear Transmutation and Oxidation Effects Using High-Energy and Low-Fluence Helium Ion Beam Irradiation
Source: Polymers (Basel). 2020 Nov 24;12(12):2770. doi: 10.3390/polym12122770 (PMC7761050; doi:10.3390/polym12122770)
Supplement: Supplementary file 1 [file polymers-12-02770-s001.pdf]

Supplementary Materials

for

Hydrophilicity improvement of polymer surfaces induced by simultaneous nuclear transmutation and oxidation effects using high-energy and low-fluence helium ion beam irradiation

Jung Woo Kim[1,2], Seung Hwa Yoo[3], Young Bae Kong[1], Sung Oh Cho[2], Eun Je Lee[1*]*

[1]Advanced Radiation Technology Institute, Korea Atomic Energy Research Institute, 29 Geumgu-gil, Jeongeup-si, Jeollabuk-do 56212, Republic of Korea

[2]Department of Nuclear and Quantum Engineering, Korea Advanced Institute of Science and Technology, 291 Daehak-ro, Yuseong-gu, Daejeon, 34141 Republic of Korea

[3]Department of Quantum System Engineering, College of Engineering, Jeonbuk National University, Jeonju, 567 Republic of Korea

[*] Corresponding author. E-mail: leeeunje@kaeri.re.kr (Eun Je Lee)
[*] Corresponding author. E-mail: seunghwayoo@jbnu.ac.kr (Seung Hwa Yoo)

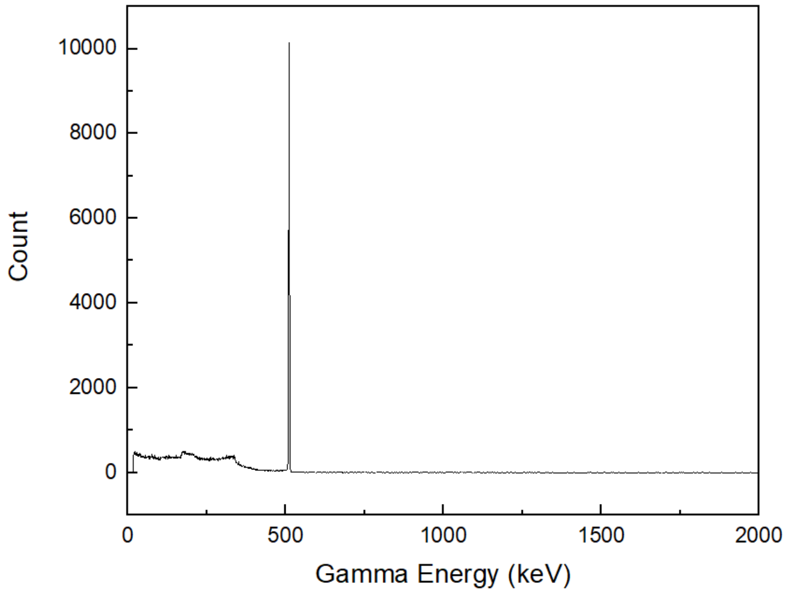

Figure S1. Measured gamma spectrum of polymer irradiated with 20 MeV helium ion beam.

Figure S1 shows the gamma spectrum of the irradiated polymer. A strong single peak centered at 511 keV was observed, which corresponds to the annihilation radiation (two 511 keV photons emitted in opposite directions) originating from the recombination of an electron (β^-) and positron (β^+). As discussed in the manuscript, positron-emitting nuclei (O-15) are formed by nuclear reaction (#1 and #5), and this radioisotope decays to a stable isotope (N-15). This decay process generates β^+, which subsequently recombine with electrons to finally emit two 511 keV photons. Therefore, the measured gamma spectrum is strong evidence that C-12 was transformed to O-15 and subsequently to N-15.
